# SELF-REPORTED QUALITY OF LIFE, IMPULSIVITY AND NON-ADHERENCE TO IMMUNOSUPPRESSIVE MEDICATION AFTER LIVER TRANSPLANTATION: A COHORT STUDY

**DOI:** 10.1590/S0004-2803.24612025-012

**Published:** 2025-09-05

**Authors:** Ana Paula JESUS-NUNES, Tayne M MOREIRA, Mychelle MORAIS-DE-JESUS, Raymundo PARANÁ, Liliane LINS-KUSTERER, Lucas C Quarantini

**Affiliations:** 1 Universidade Federal da Bahia, Faculdade de Medicina, Programa de Pós-graduação em Medicina e Saúde, Salvador, BA. Brasil.; 2 Universidade Federal da Bahia, Hospital Universitário Professor Edgard Santos, Laboratório de Neuropsicofarmacologia, Serviço de Psiquiatria do Salvador, BA, Brasil.; 3 Universidade Federal da Bahia, Salvador, BA, Brasil.

**Keywords:** Liver transplantation, medication nonadherence, transplant psychosocial evaluation, health-related quality of life, Transplante de fígado, não adesão à medicação, avaliação psicossocial de transplante, qualidade de vida relacionada à saúde

## Abstract

**Objective:**

Identify psychosocial risk factors for non-adherence to medication following liver transplantation.

**Methods:**

We used the Medication Level Variability Index (MLVI) for the assessment of adherence in 52 subjects selected for a pre-transplant liver procedure and monitored them for 6 months following transplantation. Patients were divided into exposed and non-exposed groups according to adherence, and each group was analyzed using psychosocial variables: demographic characteristics, quality of life, impulsivity, resilience, anxiety and depression.

**Results:**

Patients with non-adherence had lower scores in the SF-36v2 domains and in the components of physical and mental health, with significant differences in the physical functioning (*P*=0.03) and physical health component (*P*=0.03) domains. In addition, non-adherent patients showed higher levels of impulsiveness (*P*=0.04) and 44.2% of the non-adherent patients being men (*P*=0.04).

**Conclusion:**

Physical functioning and summary of the physical components of quality of life, impulsivity and male gender were associated with low adherence to medication.

## HIGHLIGHTS

• Psychosocial and behavior variables influence adherence in post-liver transplantation subjects.

• Quality of life domain scoreswere lower in individuals with non-adherence.

• Understanding the factors that promote poor adherence may be helpful in guiding psychosocial interventions.

## RESUMO

**Objetivo:**

Identificar fatores de risco psicossociais para a não adesão medicamentosa após transplante de fígado.

**Métodos:**

Utilizamos o Medication Level Variability Index (MLVI) para avaliar a adesão em 52 indivíduos elegíveis no pré-transplante hepático e foram monitorados por seis meses após o transplante. Os pacientes foram divididos em grupos expostos e não expostos de acordo com a adesão, e cada grupo foi analisado usando variáveis psicossociais: características demográficas, qualidade de vida (SF-36v2), impulsividade, resiliência, ansiedade e depressão.

**Resultados:**

Pacientes com não adesão apresentaram menores escores nos domínios do SF-36v2 e nos componentes de saúde física e mental, com diferenças significativas nos domínios de funcionamento físico (*P*=0,03) e componente de saúde física (*P*=0,03). Além disso, os pacientes não aderentes apresentaram maiores níveis de impulsividade (*P*=0,04) e 44,2% dos pacientes não aderentes eram homens (*P*=0,04).

**Conclusão:**

Funcionamento físico e resumo dos componentes físicos da qualidade de vida, impulsividade e gênero masculino foram associados à baixa adesão à medicação.

## INTRODUCTION

Liver transplantation is an expensive, but effective treatment for patients with end-stage liver disease. The number of donated organs is scarce, and the gap between availability and demand, has been growing annually, thus decreasing patients’ survival chances^1^.

Adherence to a medical prescription is a behavioral measure, characterized by correct intake of medications and fulfillment of the prescribed and agreed recommendations of the care provider^2^. The regular use of immunosuppressants is one of the major behavior challenges to successful transplantation and is crucial for short and long-term outcomes. Poor adherence to medication may be responsible for one in every ten deaths after liver transplantation^3^. However, between 15% and 40% of patients present non-adherence to immunosuppressive medication following liver transplantation, with obvious negative outcomes. Late acute rejection to liver transplantation is four times more likely in non-adherent patients^4^. Previous studies suggest several characteristics for non-adherence post-liver transplantation: high cost of medication, patient’s conviction that the medication is harmful, age under 40 years, having completed five or more years after transplantation, psychiatric disorders, and medication side effects^5^.

Previous studies involving kidney transplants suggest that less perceived social support, lower mental, and higher physical functioning quality of life can be risk-factors for non-adherence to immunosuppressive medication^6^. Although researchers have previously proposed certain risk factors, especially socio-demographic factors, there has been little research concerning psychosocial variables in the context of liver transplantation. The proposed associations are notably heterogeneous, due to the different ways non-adherence is evaluated, as well as the variations in sample size and the cultural and socio-economic conditions of the evaluated patients^5^.

Although previous studies have found associations between sociodemographic and psychosocial variables and therapeutic adherence, such findings are inconsistent, and discussions concerning this topic are ongoing. A recent study found that none of the psychosocial variables explored in pre-transplantation evaluation (such as psychiatric comorbidity, substance abuse and lack of social support) predicted non-adherence in liver transplant recipients^7^. There is also evidence of a relationship between impulsivity and low adherence. In a study of HIV patients, non-planning impulsiveness was associated with non-adherence^8^.

Considering the limited availability of organs for liver transplantation, it is important to identify factors that correlate to non-adherence. This identification may help orientate psychosocial interventions in the follow-up procedure for these patients and ultimately improve success rates. In this study, we aimed to identify psychosocial risk factors for non-adherence in post-liver transplantation.

## METHODS

### Design, sample and setting

A cohort study was conducted at a University Hospital of the Federal University of Bahia, recruiting patients between September 1, 2014 and June 20, 2015. Patients aged ≥18 years who had undergone pre-transplant clinically evaluation (2010 to 2014) and who had received a liver transplant, using immunosuppression schemes with tacrolimus, were included. To avoid the high variability in the concentration of the immunosuppressive drug during the initial dose adjustment period, we only considered samples taken 6 months after transplantation. Patients with less than 4 measures of tacrolimus levels in their blood were excluded from the analyses. All participants agreed to sign a consent form.

### Outcomes

The outcome of non-adherence was defined according to the Medication Level Variability Index (MLVI)^9^ ^-^ ^11^. The MLVI is defined as the standard deviation (SD) of a set of at least three tacrolimus blood levels for each patient^11^. In most medical centers, tacrolimus levels are measured at least quarterly. We then calculated the SD for serum levels of the immunosuppressant with at least four measures per patient^12^. Patients with less than four serum measurements were excluded. We used an MLVI cut-off of 2.5. Patients were divided into exposed and non-exposed groups according to adherence, and each group was analyzed using psychosocial variables.

### Variables of interest

Age, sex, and marital status were assessed through a socio-demographic questionnaire. Depression and anxiety were measured using the Hospital Anxiety and Depression Scale - HAD, a self-report instrument for the assessment of anxiety and depression among medically ill patients. We used the following cut-off: eitgh for anxiety and nine for depression^13^.

We measured health-related quality of life using the 36-Item Short-Form Health Survey (SF-36v2) questionnaire. SF-36v2 is a widely used instrument, containing eighth domains (role physical, bodily pain, general health, vitality, social functioning, role emotional, mental health). The higher the scores, the higher the subjective health-related quality of life. SF-36v2 domains generate a physical component summary (PCS) and a mental component summary (MCS). Scale scoring and component summaries were performed and normalized using PROCoRE v 1.3. All normalized scores were adjusted to a mean of 50 and a standard deviation of 10, enabling comparisons between domains. We used the OptumInsight Life Sciences Inc. system, license number: QM025905. This scale has been validated for Brazilian Portuguese (SF-36v2) in Brazilian HIV patients^14^.

The Barratt Impulsiveness Scale (BIS11), a 30-item self-report instrument, was used to evaluate impulsiveness^15^. The total score was analyzed regarding the following cut-off points: <52 low impulsivity, between 52 and 71 normal limit of impulsivity and >71 high impulsivity^16^. In our sample, we compared low and normal impulsivity with high impulsivity.

To measure levels of positive psychosocial adaptation to important life events, the 25-item resilience scale developed by Wagnild & Young was used^17^ ^,^ ^18^. The low and medium ratings were defined as a variable and compared with high resilience.

### Statistical analysis

For statistical analysis, we used the Statistical Package for the Social Sciences (SPSS), version 20.0. Demographics and variables of interest were reported in absolute and relative frequencies or by mean and standard deviation. Comparisons of continuous variables were carried out using the Mann-Whitney nonparametric test. Fisher’s Exact Test was used to compare categorical variables between adherent and non-adherent groups. We considered a *P*-value of less than 0.05 as statistically significant.

### Ethical considerations

This study was approved by the Institutional Review Board (MCO-UFBA - protocol number 14/2002). It follows the Declaration of Helsinki 2013 and the Brazilian National Health Council Resolution 499/12. The researchers ensured that the documents would be kept confidential.

### Support

This project was partially supported by the National Council of Technological and Scientific Development (CNPq): 462014/2014-2 - Edital Universal MCT/CNPQ 2014. This study was financed in part by the Coordenação de Aperfeiçoamento de Pessoal de Nível Superior - Brasil (CAPES) - Finance Code 001. The funders had no role in study design, data collection and analysis, decision to publish, or preparation of the manuscript.

## RESULTS

The study sample consisted of 52 patients. 61.5% of the patients were non-adherent. Table 1 shows baseline demographic characteristics of the study sample. No differences were noted in age or marital status. The mean age was 53.8±7.0 years; patients were mostly male (80.8%) and married or in stable relationships (76.0%). Of the 80.8% of male participants in the study, 44.2% of them were non-adherent, a statistically significant higher proportion than females. The majority of our population (76.2%) had fewer than twelve years of formal education, that is, they had no college degree.

**TABLE 1:** Demographic characteristics of adherent and non-adherent patients in post-liver transplantation patients.

	Total (%)	Adherent (%)	Non-adherent (%)	P value
Age mean ±SD	53.8±7.0	53.9±7	51±13.2	0.97*
Sex (N=52)				0.04**
Female	10 (19.2)	1 (1.9)	9 (17.3)	
Male	42 (80.8)	19 (36.6)	23 (44.2)	
Marital status (N=50)				0.57**
Married/stable relationship	38 (76.0)	15 (30.0)	23 (46.0)	
Single/widowed/divorced	12 (24.0)	5 (10.0)	7 (12.0)	
Education (N=42)				0.165**
College graduate/Some College education	10 (23.8)	2 (4.8)	8 (19.0)	
<High school	32 (76.2)	14 (33.3)	18 (42.9)	

*Mann-Whitney test; **Fisher exact test. SD: standard deviation.

Table 2 shows the psychosocial variables of the sample at baseline. In our sample, 11.8% of patients exhibited high impulsiveness. We observed that non-adherent patients showed higher levels of impulsiveness (*P*=0.04). Most patients presented high/moderate resilience (81.6%), which is consistent with the effective confrontation of adversities inherent to the transplantation process. There was a low prevalence of depression in our sample (only 4%). The frequency of anxiety was moderate (14.3%).

**TABLE 2:** Psychosocial variables according to therapeutic adherence in post-liver transplantation patients.

	Total (%)	Adherent (%)	Non-adherent (%)	*P* value*
BIS-11 (N=51)				0.04
High impulsiveness	6 (11.8)	0 (0.0)	6 (11.8)	
Normal/ low impulsiveness	45 (88.2)	20 (39.2)	25 (49.0)	
Resilience Scale (N=49)				0.49
High/Moderate Resilience	40 (81.6)	15 (30.6)	25 (51.0)	
Low resilience	9 (18.4)	4 (8.2)	5 (10.2)	
HADS anxiety (N=49)				0.49
With anxiety	7 (14.3)	2 (4.1)	5 (10.2)	
Without anxiety	42 (85.7)	16 (32.7)	26 (53.0)	
HADS depression (N=50)				0.60
With depression	2 (4.0)	1 (2.0)	1 (2.0)	
Without depression	48 (96.0)	17 (34.0)	31 (62.0)	

*Fisher exact test.

SF-36v2 scores between Adherent and Non-adherent patients at baseline are shown in Table 3. All SF-36v2 domains and the physical and mental health components were systematically lower in individuals with non-adherence, with statistically significant differences in the physical functioning (*P*=0.03), and physical health component (*P*=0.03) domains.

**TABLE 3:** SF-36v2 scores between adherent and non-adherent post-liver transplantation patients.

SF-36v2	Total (n=52) M±SD	Cronbach’s alpha	Adherent (n=20) M±SD	Non-adherent (n=32) M±SD	*P* value*
Physical functioning	47.4±9.7	0.87	50.7±9.1	45.5±9.6	0.03
Role-physical	36.4±15.4	0.89	41.5±15.5	33.3±14.7	0.08
Bodily pain	51.4±10.6	0.71	54.2±8.9	50.0±11.3	0.26
General health	46.1±7.7	0.70	47.2±6.5	45.6±8.4	0.71
Vitality	53.4±11.5	0.78	57.3±9.8	51.2±12.2	0.10
Social functioning	43.7±8.7	0.60	44.4±9.3	43.2±8.0	0.71
Role-emotional	50.5±11.3	0.65	53.2±8.8	49.6±11.7	0.27
Mental health	53.4±8.8	0.74	55.5±6.0	53.4±9.6	0.77
Physical component summary	42.7±10.7		46.4±10.7	40.5±10.2	0.03
Mental component summary	53.8±9.7		54.8±9.3	53.1±10.0	0.71

*Mann-Whitney U Test. SF-36v2: 36-Item Short Form Health Survey questionnaire, version 2.0.

All mean quality of life scores relating to the health of individuals with adherence were within the expected range (50±10).

Although there is no statistically significant association in respect of the role-physical domain, the results indicate that the average score of non-adherent patients (33.3±14.7) is well below the average of adherent patients.

The patients in our sample showed compromised physical health, with lower scores than in the domains that assessed mental health and in which they did not perform well.

## DISCUSSION

The aim of this study was to identify psychosocial variables that influence the adherence to medication in post-liver transplantation subjects. Our sample had a 61.5% non-adherence to immunosuppressive drugs profile. This percentage was similar to a study in which 50% of patients did not adhere to tacrolimus^7^, although most previous studies, indicate that 15% to 40% do not adhere to immunosuppressive drugs^9^. In other reports involving self-reported non-adherence, frequencies of non-adherence were also high (62%)^9^.

Differences in the sociodemographic characteristics of our population may justify the high prevalence of non-adherence, since there seems to be an association between level of education and poor adherence to immunosuppressive medication^2^ ^,^ ^5^ ^,^ ^19^. Patients with improved scores after an educational counseling program (S-SMAOT) had a significant correlation with adherence to immunosuppressive therapy, suggesting that a better understanding of the treatment enhances adherence^20^. In Brazil, around 18% of 18-64 year old adults have access to higher education, a rate similar to our sample, which showed that 23.8% of the patients had attended college. This rate is much lower than in other countries in Latin America and less than half that of other countries in the Organization for Economic Cooperation and Development (OECD), where the average rate for entering higher education is 39%^21^.

In a previous study, Male gender was a significant predictor of non-adherence (Colmenero et al. 2024)^22^ ^,^ ^23^. This data was also observed in our sample, with 44.2% of the non-adherent patients being men (*P*=0.04), compared to 17.3% women.

Non-adherence in patients with chronic diseases is not only associated with being a burden on health services, but is also directly associated with having greater functional impairment and, consequently, a worse quality of life^24^. Evaluation of psychosocial variables is a useful auxiliary tool in the selection process of patients eligible for liver transplantation^25^. It is expected that patients undergoing transplantation will improve their quality of life^24^.

In our study, we observed that the lowest levels of pre-transplant physical functioning are associated with poor adherence (*P*=0.03). Additionally, we observed the same association when scores were summarized in the Physical Component Summary of the SF-36v2 (*P*=0.03). We hypothesized that patients with worse physical functioning are more likely to have difficulties in performing their daily tasks, requiring additional assistance to perform simple self-care activities, such as administering their medication.

Our data demonstrates that non-adherent patients had normal or elevated impulsivity. These data are consistent with a study of HIV patients which demonstrated that non-adherence was associated with non-planning impulsivity, defined as a current action that is not sensitive to future consequences^8^ and illustrated in a study with euthymic bipolar disorder patients^26^. One study with patients from drug and alcohol rehabilitation units found a negative correlation between the motor dimension of impulsivity and compliance^27^. The intervention for impulsive behavior is self-control, learned through exposure to effort, reward discrimination, reward grouping, reinforcement interval schedules, and impulse control training^28^. Therefore, impulsivity should be considered when planning treatment.

Psychiatric comorbidities such as depression and anxiety, usually associated with the lowest rates of adherence, showed no differences between the two groups^5^ ^,^ ^23^. The low prevalence of anxiety and depression in our sample may have contributed to the absence of differences found between the two groups. Patients who have a history of psychiatric disorder and have undergone appropriate treatment have less psychiatric decompensation after transplantation^25^.

One of the strengths of this study is the use of serum immunosuppressant dosage with at least four measures, compared to self-report measures. As it is an objective measure of evaluation, data omittance by patients is avoided. This prospective cohort study also covers a wide range of behavioral and psychosocial variables that may influence adherence.

Our study has certain limitations. Firstly, our sample is small and presents a higher proportion of males to females, which can also be seen in other studies^23^. Secondly, even though there is no definitive benchmark measure for adherence, we did not use any secondary assessment^5^. Subjective methods, such as self-reporting, are unreliable while other objective methods, such as counting pills or electronic monitoring, are expensive and/or unavailable. Therefore, there is an increasing interest in identifying simple methods that could be used in consultations to adequately identify a patient’s adherence pattern and thus incorporated into a care package^6^. It is important to emphasize that we used a simple and inexpensive, yet reliable, method.

In conclusion, we identified that quality of life, specifically physical functioning and physical component summary, impulsivity, and male gender, were associated with non-adherence behavior. The identification of variables related to immunosuppressive drug adherence could help healthcare professionals develop strategies that promote better adherence. Interventions based on behavioral therapies and mindfulness contribute to improving adherence^8^. Finally, it is worth stating that further studies to identify psychosocial variables that interfere with medication adherence after liver transplantation are needed.
